# Ezetimibe Enhances Lipid Droplet and Mitochondria Contact Formation, Improving Fatty Acid Transfer and Reducing Lipotoxicity in Alport Syndrome Podocytes

**DOI:** 10.3390/ijms252313134

**Published:** 2024-12-06

**Authors:** Jin-Ju Kim, Eun-Jeong Yang, Judith Molina David, Sunjoo Cho, Maria Ficarella, Nils Pape, Josephin Elizabeth Schiffer, Rachel Njeim, Stephanie S. Kim, Claudia Lo Re, Antonio Fontanella, Maria Kaber, Alexis Sloan, Sandra Merscher, Alessia Fornoni

**Affiliations:** 1 Katz Family Division of Nephrology and Hypertension, Department of Medicine, University of Miami Miller School of Medicine, Miami, FL 33136, USA; j.molina4@med.miami.edu (J.M.D.); s.cho6@med.miami.edu (S.C.); mxf1614@miami.edu (M.F.); nilspapenp@gmail.com (N.P.); josischiffer@gmail.com (J.E.S.); rxn404@miami.edu (R.N.); ssk781@miami.edu (S.S.K.); cxl1870@miami.edu (C.L.R.); axf870@med.miami.edu (A.F.); mek2030@miami.edu (M.K.); asloan@med.miami.edu (A.S.); smerscher@med.miami.edu (S.M.); 2 Peggy and Harold Katz Family Drug Discovery Center, University of Miami Miller School of Medicine, Miami, FL 33136, USA; 3 Institute for Immunology and Immunological Diseases, Yonsei University College of Medicine, Seoul 03722, Republic of Korea; yej5644@yuhs.ac; 4 Unit of Nephrology and Dialysis, Department of Clinical and Experimental Medicine, A.O.U “G. Martino”, University of Messina, 98122 Messina, Italy

**Keywords:** lipid droplet, fatty acid, lipotoxicity, Alport syndrome podocytes

## Abstract

Mitochondrial dysfunction is a critical factor in the pathogenesis of Alport syndrome (AS), contributing to podocyte injury and disease progression. Ezetimibe, a lipid-lowering drug, is known to inhibit cholesterol and fatty acid uptake and to reduce triglyceride content in the kidney cortex of mice with AS. However, its effects on lipid droplet (LD) utilization by mitochondria have not been explored. Transmission electron microscopy (TEM) and mitochondrial functional assays (ATP production, mitochondrial membrane potential, and citrate synthase activity) were used to investigate the impact of ezetimibe on LD–mitochondria contact formation and mitochondrial function in *Col4a3*KO (AS) and wildtype (WT) podocytes. TEM analysis revealed significant mitochondrial abnormalities in AS podocytes, including swollen mitochondria and reduced cristae density, while mitochondrial function assays showed decreased ATP production and lowered mitochondrial membrane potential. AS podocytes also demonstrated a higher content of LD but with reduced LD–mitochondria contact sites. Ezetimibe treatment significantly increased the number of LD–mitochondria contact sites, enhanced fatty acid transfer efficiency, and reduced intracellular lipid accumulation. These changes were associated with a marked reduction in the markers of lipotoxicity, such as apoptosis and oxidative stress. Mitochondrial function was significantly improved, evidenced by increased basal respiration, ATP production, maximal respiration capacity, and the restoration of mitochondrial membrane potential. Additionally, mitochondrial swelling was significantly reduced in ezetimibe-treated AS podocytes. Our findings reveal a novel role for ezetimibe in enhancing LD–mitochondria contact formation, leading to more efficient fatty acid transfer, reduced lipotoxicity, and improved mitochondrial function in AS podocytes. These results suggest that ezetimibe could be a promising therapeutic agent for treating mitochondrial dysfunction and lipid metabolism abnormalities in AS.

## 1. Introduction

Alport syndrome (AS) is a hereditary disease of the glomerular basement membrane (GBM) caused by mutations in the genes coding for different chains of collagen type IV (Col IV) [1,2,3]. Mutations in the α3, α4, or α5 chains of Col IV disturb the normal formation of the basement membranes in the kidney [1], eye [4], and inner ear [5]. In the kidney, the integrity of the glomerular filtration barrier is disrupted, initially resulting in GBM ultrastructural changes, followed by progressive renal failure [6]. Patients with AS develop end-stage renal disease during adolescence or early adulthood [7]. Yet, no specific treatment is currently available for AS, and ramipril represents the only standard of care based on retrospective studies [8] and supported by a recent phase-two study in adolescents affected by AS [9].

The kidney glomerulus is a highly specialized structure that ensures the selective ultrafiltration of plasma so that essential proteins are retained in the blood [10]. Podocytes are glomerular epithelial cells with numerous foot processes bridged by a 40 nm wide extracellular structure known as the slit diaphragm [11,12]. The interaction of podocytes with the GBM is crucial to maintain an intact glomerular filtration barrier [13]. However, how an altered GBM affects podocyte structure, function, and metabolism remains to be established. Just like for most glomerular disorders, podocyte injury is an important feature of AS, and a reduction in the number of podocytes is associated with disease progression in patients with AS [14]. Thus, proper podocyte function is of critical importance for sustained function of the glomerular filter.

Renal lipotoxicity, as reported by others and us, is an important contributor to the pathogenesis of several forms of kidney disease, including AS [15,16]. In particular, we demonstrated that lipotoxicity in terminally differentiated cells such as podocytes is a key determinant of cell injury [16,17,18]. Both impaired cholesterol efflux [16,17,19] and altered free fatty acid (FFA) metabolism in podocytes play a critical pathogenic role in diabetic kidney disease [20] as well as in kidney diseases of non-metabolic origin such as AS [15]. We and others have previously reported that altered lipid metabolism occurs in glomeruli and tubules from *Col4a3* KO mice, a mouse model for AS, and contributes to disease progression [15,21]. Lipid droplets (LDs) share the same structure across cell types and are mainly compromised of a neutralized form of lipids such as triglycerides (TGs) and cholesterol esters to protect against lipotoxicity, shielded by proteins and a phospholipid monolayer [22,23,24]. While the impairment of cholesterol efflux is not sufficient to cause podocyte apoptosis [16], podocytes are highly susceptible to apoptosis caused by FFA [25]. However, the detailed mechanism by which FFAs contribute to podocyte injury in AS remains to be elucidated.

We previously reported that an abnormal GBM, as observed in AS, alters podocyte discoidin domain receptor 1 (DDR1) signaling, leading to elevated FFA uptake, TG accumulation, and podocyte lipotoxicity [26]. The increased level of FFA uptake is due to DDR1-mediated cluster of differentiation 36 (CD36) activity. Ezetimibe, which is an FDA-approved lipid-lowering agent [27,28,29], reduces FFA uptake by interfering with the DDR1 and CD36 interaction [26]. We previously demonstrated that, unexpectedly, ezetimibe treatment reduces TG levels but not cholesterol levels in the progression of AS [26]. However, the downstream mechanism by which TG accumulation injures podocytes remains to established.

TG metabolism and cellular energy homeostasis are mediated through the lipolysis of TG stored in LDs, leading to the release of FFA [30]. Although TG lipolysis provides a convenient source of cellular fuel for energy production, excessive lipolysis can contribute to the buildup of toxic lipid intermediates and/or oxidized lipids [31,32]. In patients with non-alcoholic fatty liver disease, for example, excessive FFA catabolism resulting from the excessive lipolysis of TG is a major contributor to cellular lipotoxicity [33]. When cells need energy, TGs stored in LD are hydrolyzed into FFAs and consumed to produce ATP [34,35,36]. LD–mitochondria contact sites are crucial for the efficient transfer of FFA [36] from LD to mitochondria, which is necessary to maintain proper cellular energy homeostasis and proper lipid metabolism. Failure to establish proper LD–mitochondria contact sites can lead to the accumulation of FFAs in the cytosol, causing mitochondrial dysfunction and further exacerbating cellular injury [36,37].

Although mitochondrial dysfunction is a critical factor in the pathogenesis of various kidney diseases [17,38], it is unknown whether the mitochondrial dysfunction contributes to the progression of AS in an experimental model. Thus, in this report, we first describe mitochondrial dysfunction in the AS. Second, we demonstrate the reduction in contact formation between LD and mitochondria-mediated inefficient FA transfer as one of the main causes of mitochondrial dysfunction and podocyte injury in AS. Third, we report for the first time that ezetimibe, the lipid-lowering agent, has a role in enhancing LD–mitochondria contact formation, preventing lipotoxicity.

## 2. Results

### 2.1. Increased TG Lipolysis in Immortalized AS Podocytes Is Associated with Podocyte Injury Due to Inefficient FA Transfer Leading to Mitochondrial Dysfunction

**Figure 1:**
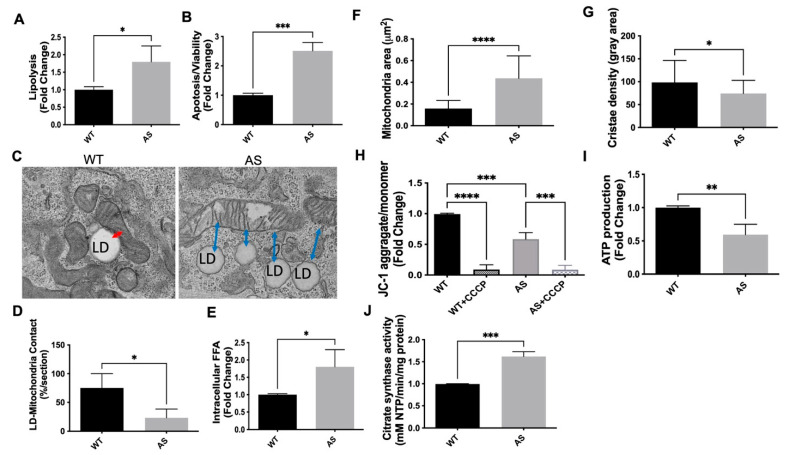
Increased TG lipolysis in immortalized AS podocytes is associated with mitochondrial dysfunction and podocyte injury due to inefficient FA transfer. (**A**) Lipolysis assay demonstrating significantly increased lipolysis in immortalized AS podocytes when compared to immortalized WT podocytes (n = 3, * *p* < 0.05, *t*-test). (**B**) Apoptosis increases in immortalized AS podocytes when compared to immortalized WT podocytes (n = 3–4, *** *p* < 0.001, *t*-test). (**C**,**D**) TEM image analysis demonstrates lower mitochondria and LD interaction in immortalized AS podocytes compared to immortalized WT podocytes; red arrow indicates LD contact with mitochondria, and blue arrow indicates distance between LD and mitochondria (**C**), quantified (**D**) (* *p* < 0.05, *t*-test). (**E**) Intracellular FFA are increased in immortalized AS podocytes when compared to immortalized WT podocytes (n = 3, * *p* < 0.05, *t*-test). (**F**,**G**) Mitochondrial area (**F**) and cristae density (**G**) of the podocytes in the TEM images were quantified by image J 1.54f. (* *p* < 0.05, **** *p* < 0.01, *t*-test). (**H**) Mitochondrial membrane potential (MMP) measured by aggregate/monomeric JC-1 ratio, demonstrating decreased MMP (n = 3, *** *p* < 0.001, **** *p* < 0.0001 one-way ANOVA). (**I**) ATP production was measured by bioluminescence (n = 3, ** *p* < 0.01 *t*-test). (**J**) Citrate synthase activity determined by the rate of absorbance gain at 412 nm normalized to protein concentration (n = 3, *** *p* < 0.001 *t*-test).

We previously reported increased TG accumulation in immortalized AS podocytes. To determine if increased TG accumulation in AS podocytes is associated with increased TG lipolysis and podocyte injury, lipolysis and apoptosis were investigated. Our data show that TG lipolysis (Figure 1A,B) are significantly increased in immortalized AS when compared to immortalized WT podocytes. Intracellular FFA levels were measured in immortalized WT and AS podocytes. We found that FFA levels were increased in AS podocytes when compared to WT podocytes (Figure 1E), further supporting that lipolysis is increased in AS podocytes. The coordination of LD lipolysis and mitochondrial metabolism is critical for the removal of toxic FFA species and the prevention of lipotoxicity. LD–mitochondrial contact formation is crucial for efficient FA transfer from LDs to mitochondria and ATP production to prevent FA cytotoxicity [39,40,41]. We next examined if the increased cytosolic FFA pool in AS could be caused by the impaired formation of LD–mitochondrial contacts. The TEM image analysis of immortalized podocytes showed a significant reduction in LD-mitochondrial contact formation in AS podocytes when compared to WT podocytes (Figure 1C,D). This observation suggests that FAs produced due to increased TG lipolysis (Figure 1A) in AS podocytes cannot be efficiently transferred and utilized by mitochondria, thus contributing to lipotoxicity. Additionally, we observed alterations in mitochondrial morphology including decreased cristae density and increased mitochondrial swelling (Figure 1F,G), as well as a reduction in mitochondrial membrane potential (Figure 1H) and ATP production (Figure 1I), potentially caused by increased FFA [42], suggesting mitochondrial dysfunction. Lastly, we found increased mitochondrial biogenesis (Figure 1J), a possible compensatory cellular response to mitochondrial dysfunction. Taken together, our data suggest that in immortalized AS podocytes, increased cytoplasmic FFA are not efficiently transferred from LDs to mitochondria due to impaired LD–mitochondria contact formation, thereby contributing to mitochondrial dysfunction and podocyte injury.

### 2.2. Ezetimibe Improves Lipid Droplet and Mitochondria Contact Formations

**Figure 2:**
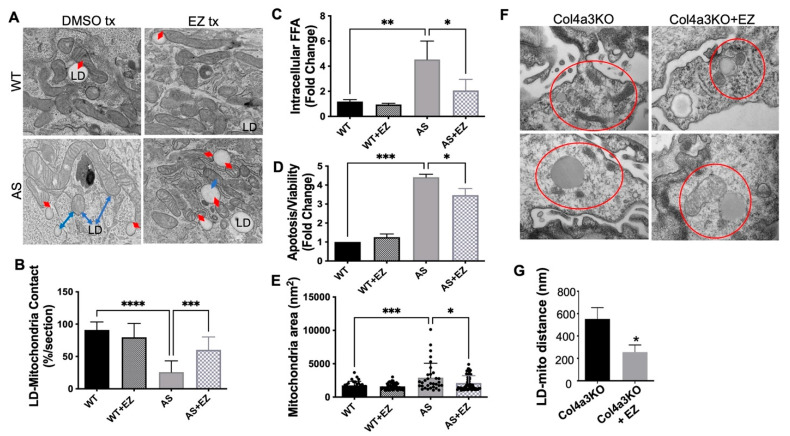
Ezetimibe improves LD–mitochondria contact formation and protects from immortalized podocyte injury. (**A**,**B**) TEM analysis demonstrating increased LD–mitochondria contact in immortalized AS podocytes treated with ezetimibe compared to untreated AS podocytes; red arrows point to LD and mitochondria contact, and blue arrows indicate the distance between LD and mitochondria (**A**), and the quantification (**B**) of LD and mitochondria contact formation (n = 3 mice per group, *** *p* < 0.001 **** *p* < 0.0001, one-way ANOVA). (**C**) The intracellular FFA quantification assay demonstrates that ezetimibe treatment reduces FFA in immortalized AS podocytes compared to untreated AS podocytes (n = 3, * *p* < 0.05, ** *p* < 0.01) (**D**) The apoptosis assay demonstrates that ezetimibe treatment of immortalized AS podocytes reduces apoptosis compared to untreated AS podocytes (n = 3–4, * *p* < 0.05, *** *p* < 0.001, one-way ANOVA). (**E**) The quantified mitochondrial area of the TEM image analysis demonstrates that ezetimibe treatment on immortalized AS podocytes prevents mitochondria swelling (n = 3, * *p* < 0.05, *** *p* < 0.001). (**F**,**G**) TEM image of Col4a3KO mice and ezetimibe-treated *Col4a3*KO mice, indicating that the ezetimibe treatment reduces the distance of LD and mitochondria (**F**) and the bar graph of the quantification (**G**) (n = 3, * *p* < 0.05).

Ezetimibe is an FDA-approved drug for hypercholesterolemia. Besides its ability to prevent cholesterol adsorption, experimental data from others suggested that it can affect FFA uptake in hepatocytes [43]. We previously have reported that ezetimibe inhibits FFA uptake via the blocking of the interactions of CD36 and DDR1. We recently demonstrated that ezetimibe treatment of *Col4a3* KO mice, an experimental mouse model of AS, improves kidney function [26]. Here, we show that ezetimibe improves LD–mitochondria contact formation in immortalized AS podocytes treated with ezetimibe compared to untreated AS podocytes (Figure 2A,B). Enhanced LD–mitochondria contact formation was observed in ezetimibe-treated AS podocytes (Figure 2A,B) and in the glomeruli of *Col4a3*KO mice (Figure 2F,G), suggesting that ezetimibe treatment results in a more efficient fatty acid (FA) transfer from LDs to mitochondria and thereby protects against podocyte injury. To test this hypothesis, we measured the levels of FFAs and apoptosis in ezetimibe-treated immortalized AS podocytes. As expected, ezetimibe treatment significantly reduced the FFA level (Figure 2C), podocyte injury (Figure 2D), and mitochondrial swelling (Figure 2E) of AS podocytes. We also found a significant increase in the rate of FA transfer to mitochondria in ezetimibe-treated immortalized podocytes, which was accompanied by the increased LD–mitochondria contact formation. These findings suggest that by promoting efficient FA transfer, ezetimibe mitigates the lipotoxic effects of accumulated FAs.

## 3. Discussion

Our findings extend previous research, demonstrating that podocytes undergo metabolic shifts in disease [44] and that impaired TG and FA metabolism, particularly the reduced formation of LD and mitochondria contact sites, plays a critical role in podocyte injury in AS. These contact sites are essential for efficient FA transfer from LDs to mitochondria, where FAs are oxidized to produce ATP [35,36,45]. The impaired contact formation observed in AS podocytes leads to inefficient FA utilization, resulting in elevated cytosolic FFAs and mitochondrial dysfunction, which exacerbates podocyte injury (Figure 1). Our findings demonstrate that lipid droplet (LD) underutilization is a specific and critical pathological feature in AS podocytes. Transmission electron microscopy (TEM) revealed a marked reduction in LD–mitochondria contact formation in AS podocytes compared to wild-type (WT) controls, indicating that FAs generated through lipolysis cannot be effectively transferred to mitochondria. This inefficiency exacerbates cytosolic FFA accumulation, contributing to mitochondrial dysfunction, including decreased ATP production, increased mitochondrial swelling, and reduced cristae density (Figure 1). These findings are consistent with previous studies linking LD–mitochondria interactions to energy production and lipotoxicity prevention in podocytes. The enhancement of LD–mitochondria contact sites by ezetimibe observed in our study further supports the central role of LD underutilization in AS pathology and highlights its potential as a therapeutic target.

In a previous study, we demonstrated for the first time that ezetimibe, a lipid-lowering agent, reduces FFA uptake via the inhibition of the CD36 activity [26]. Additionally, we analyzed NPC1L1 expression in WT and AS podocytes and found that while NPC1L1 is detectable, its expression is relatively low compared to that in the small intestine, where its role in cholesterol metabolism is well established [46] (Supplementary Figure S2). This observation suggests that the ability of ezetimibe to reduce triglyceride accumulation in AS podocytes is not primarily due to its effects on NPC1L1-mediated cholesterol absorption but rather through its inhibition of DDR1-CD36 interactions and subsequent modulation of fatty acid metabolism. In this study, we provide evidence that ezetimibe also enhances LD–mitochondrial contact formation, which leads to improved FA transfer efficiency, reduces FFA accumulation, and mitigates podocyte apoptosis (Figure 2). While ezetimibe effectively lowers TG levels, its lack of effect on cholesterol levels indicates distinct regulatory pathways for these types of lipids in AS [26]. Furthermore, statins and ezetimibe have been studied for their potential nephroprotective effects [47]. While statins are recommended for CKD patients to reduce cardiovascular risk, their impact on slowing kidney disease progression remains inconclusive [48]. Preclinical studies have shown that statins may benefit podocyte function in *Col4a3−/−* mice, but these findings have yet to be validated in AS patients. Ezetimibe, on the other hand, has been shown to partially protect against albuminuria and CKD progression in *Col4a3−/−* mice by reducing CD36-dependent fatty acid uptake and triglyceride content [26]. However, whether these effects translate to AS patients remains to be established. Recent studies in diabetes and CKD models have demonstrated that ezetimibe reduces kidney parenchyma fat content, supporting its potential application in AS [49]. Our findings suggest that ezetimibe’s therapeutic potential in AS extends beyond lipid-lowering properties to include protective effects on mitochondrial function and FA metabolism. However, while we observed a significant reduction in LD–mitochondrial contact formation in AS podocytes, the precise molecular mechanisms underlying this dysfunction were not fully elucidated. Thus, it would be important to study LD-associated proteins, such as members of the perilipin family [36,41], to determine if these proteins plays a crucial role in LD–mitochondria contact formation [24,50] and if they are regulated by ezetimibe. In addition, exploring the potential synergy between ezetimibe and other lipid-regulating agents could open new therapeutic avenues. Since distinct pathways regulate TG and cholesterol metabolism [16,17,26,51], combination therapies may offer more comprehensive protection for podocytes in AS. These findings highlight a previously unrecognized mechanism of ezetimibe action and suggest potential therapeutic benefits for AS patients by targeting mitochondrial dysfunction and lipid metabolism. To support the translational potential of our findings, we recently conducted a phase 2 study in patients with diabetes and demonstrated that ezetimibe treatment is associated with a decreased renal parenchyma fat content as assessed by MRI, thus offering a minimally invasive tool to monitor response to treatment in future clinical development [49].

The limitation of our study includes a purely in vitro design and the inability to identify the exact mechanisms responsible for the lack of LD–mitochondria contact in AS. Additionally, the protective effects of ezetimibe on the mitochondrial dysfunction in AS mice remained unclear. While this study primarily focuses on mechanistic insights at the cellular level, we recognize the limitations of the current in vivo data. Further investigations, including a comprehensive analysis of mitochondrial function and morphology in AS mouse models, are warranted to address this gap. Nevertheless, the in vitro design allowed us to better characterize the specific effect of ezetimibe on cell metabolism, and both experimental and clinical efficacies were described in parallel studies and support the clinical relevance of our findings. Additional drug development studies will be needed to develop a targeted therapeutic agent that would allow for proper LD–mitochondria contact.

## 4. Methods

### 4.1. Podocyte Cell Culture

Immortalized AS and WT podocytes were established by us previously [26,52] via breeding *Col4a3*KO mice with H-2kb-tsA58 transgenic mice purchased from Charles River (Wilmington, MA, USA, CBA/CaxC57BL/10-H-2Kb-tsA58) [53]. Immortalized cell lines were cultured at 33 °C in RPMI growth medium (containing 10% fetal bovine serum, 1% penicillin/streptomycin, 10 U/mL IFN γ) under permissive conditions. Podocyte cell lines were then thermo-shifted to 37 °C non-permissive conditions in the absence of IFNγ for 12 days of differentiation. To further characterize WT and AS podocyte cell lines, differentially expressed gene (DEG) analysis was performed on transcriptomic data from these cell lines (Supplementary Figure S1).

### 4.2. Ezetimibe Treatment

For in vitro studies, WT and AS podocyte were differentiated for 9 days and serum-starved for 12 h and treated with ezetimibe 24 µM in complete medium for 48 h.

For in vivo studies, *Col4a3* KO mice (129-*Col4a3*^tm1Dec^/J, stock number 002908, Jackson Laboratories, Bar Harbor, ME, USA) were used to evaluate the effects of ezetimibe on lipid metabolism and renal function as previously reported [26]. Ezetimibe was orally administered at a dose of 5 mg/kg body weight daily, starting at 4 weeks of age, for a duration of 4 weeks. The mice were sacrificed at 8 weeks of age. The experimental groups included WT, WT + EZ, *Col4a3*KO, and *Col4a3* KO + EZ.

### 4.3. Transmission Electron Microscopy

Kidneys from three mice per group were harvested at the time of sacrifice and transferred into a solution of 0.1 M phosphate buffer (pH 7.4) containing 4% paraformaldehyde and 1% glutaraldehyde. Podocytes were seeded in a 6-well plate with 80% confluency for differentiation. Differentiated cells were washed with 0.1 M Na-cacodylate and fixed with 2% paraformaldehyde and 2.5% glutaraldehyde in 0.1 M Sodium Cacodylate Buffer (pH 7.4). Kidney cortices and podocytes were processed for ultrastructural examination at the Electron Microscopy and Histology Core Facility at Weill Cornell Medical College. Images were taken by a digital electron microscope (JEM-1400, JEOL USA, Peabody, MA, USA) by one blinded investigator. Mitochondrial area, matrix density, and lipid droplet and mitochondria contact and distance between them were quantified using Image J by two blinded investigators. Five representative images were obtained from each group and 20 representative mitochondria were selected for analysis.

### 4.4. Mitochondrial Membrane Potential Measurement Assay

Mitochondrial membrane potential (MMP) was determined by MitoProbe™ TMRM Assay Kit (Invitrogen, ThermoFisher, Waltham, MA, USA), according to the manufacturer’s protocol. Briefly, 1 × 10^6^ differentiated podocytes were resuspended in 1 mL phosphate-buffered saline (PBS). For the control samples, 2 mL of 50 mM of CCCP was added to induce MMP depolarization. Experimental samples were incubated with 20 nM tetramethylrhodamine methyl ester (TMRM) for 30 min at 37 °C, 5% CO_2_. Data were acquired on a BD LSR Fortessa flow cytometer (BD Biosciences, Franklin Lakes, NJ, USA) with 561 nm excitation and the PE-Texas red filter. Membrane potential was calculated as the aggregate/monomer ratio.

### 4.5. ATP Determination Assay

The ATP determination assay was performed using the ATP Determination Kit (Molecular Probes, Thermo Fisher) following the manufacturer’s protocol. The production of cellular ATP was quantified using the bioluminescence assay in which luciferase converts D-luciferin in the presence of ATP. The production of light is proportional to the amount of ATP in cells. Data were normalized to cell numbers.

### 4.6. Citrate Synthase Assay

Citrate synthase assay was performed by measuring the reduction in DTNB (5,5′-dithiobis-(2-nitrobenzoic acid)) as previously described [38]. Differentiated podocytes were harvested and permeabilized by mechanical breakage (four freeze–thaw cycles), followed by resuspension in a buffer containing 225 mM mannitol, 75 mM sucrose, 10 mM Tris pH 7.2, and 0.1 mM EDTA. A total of 10 μL of cell homogenate was added to 10 mM Tris–HCl, pH 7.5 buffer containing 0.2% Triton X-100, 0.1 mM DTNB, and 0.2 mM acetyl-CoA. Baseline absorbance was measured at 412 nm. To initiate the reaction, 0.5 mM oxaloacetic acid was added, and the increase in absorbance at 412 nm was recorded for 1 min. The linear rate of increase was used to calculate citrate synthase activity as mM of NTB (nitro-thiobenzoate, ε = 13.6 mM^−1^ cm^−1^) product generated per minute and normalized to the protein concentration of the homogenate.

### 4.7. Lipolysis Assay

Lipolysis was determined using the glycerol assay kit (MAK117, Millipore Sigma, Burlington, MA, USA) according to the manufacturer’s protocol. Assay buffer, enzyme mix, ATP, and dye reagent were added to the samples and mixed on a horizontal shaker followed by incubation at room temperature in the dark for 20 min. Absorbance was measured at 570 nm using a Spectra Max i3X plate reader (Molecular Devices, San Jose, CA, USA).

### 4.8. Free Fatty Acid (FFA) Quantification Assay

FFA levels were measured in cell lysates using a commercial colorimetric assay kit (Millipore Sigma). Briefly, cells were washed with cold PBS, lysed with a lysis buffer, then centrifuged at 10,000× *g* for 10 min to remove debris. The supernatant containing the cell lysate was collected, and FFA content was quantified according to the manufacturer’s instructions. FFA standards were prepared, and the lysates were added to a 96-well plate along with the assay reagent. After 30 min of incubation at 37 °C, absorbance was measured at 570 nm using a microplate reader. FFA concentrations were determined by comparison to a standard curve, and values were normalized to total protein content in each sample, which was quantified using a bicinchoninic acid assay.

### 4.9. Statistics

For each statistical test, biological sample size (n), and *p*-value are indicated in the corresponding figure legends. All values are presented as mean ± SD. Statistical analysis was performed using Prism GraphPad 7 software. Significant outliers were determined by the GraphPad outlier calculator and excluded from further statistical analysis. Animals were grouped according to genotypes and then randomized, and investigators were blinded for the analyses. When comparing two groups, a two-tailed Student’s *t*-test was performed. Otherwise, results were analyzed using one-way ANOVA followed by Holm–Sidak’s multiple comparisons. A *p*-value less than 0.05 was considered statistically significant. Only data from independent experiments were analyzed.

## Data Availability

The bulk RNA-seq data generated from immortalized WT and AS podocytes are deposited in the NCBI GEO database under the accession number GSE274298 (https://www.ncbi.nlm.nih.gov/geo/query/acc.cgi?acc=GSE274298, accessed on 27 November 2024). The dataset is currently private as it is associated with a manuscript under revision for resubmission to Kidney International. The data will be made publicly available upon publication of the manuscript. Until then, the raw transcriptomic data are available upon reasonable request from the corresponding author. The processed transcriptomic data analysis is provided in the Supplementary Materials.

## Supplementary Materials

The following supporting information can be downloaded at: https://www.mdpi.com/article/10.3390/ijms252313134/s1.
