# Supplementary material for: Ezetimibe Enhances Lipid Droplet and Mitochondria Contact Formation, Improving Fatty Acid Transfer and Reducing Lipotoxicity in Alport Syndrome Podocytes
Source: Int J Mol Sci. 2024 Dec 6;25(23):13134. doi: 10.3390/ijms252313134 (PMC11642288; doi:10.3390/ijms252313134)
Supplement: Supplementary file 1 [file ijms-25-13134-s001.zip › ijms-3311246-supplementary.pdf]

Supplemental data Figure S1.

To characterize the WT and AS podocyte cell lines, we performed differentially expressed gene (DEG) analysis on transcriptomic data from WT and AS podocytes. This analysis identified 1,230 genes that were differentially expressed between WT and AS podocytes, as shown in the volcano plot (p-value <0.05, |FC|>2). The log2 FC indicates the mean expression level for each gene. Each dot represents one gene. Black dots represent no significant DEGs between WT and AS podocytes. Blue dots represent down-regulated genes and red dots represent up-regulated genes. Notably, Col4A3 expression (green dot) is significantly decreased in AS podocytes, while Col4A4 (orange dot) and Col4A5 (yellow dot) remain unchanged. These results indicate an abnormal production of the mature $\alpha3\alpha4\alpha5$ collagen type IV network in AS podocytes, consistent with the pathological mechanism of Alport Syndrome.

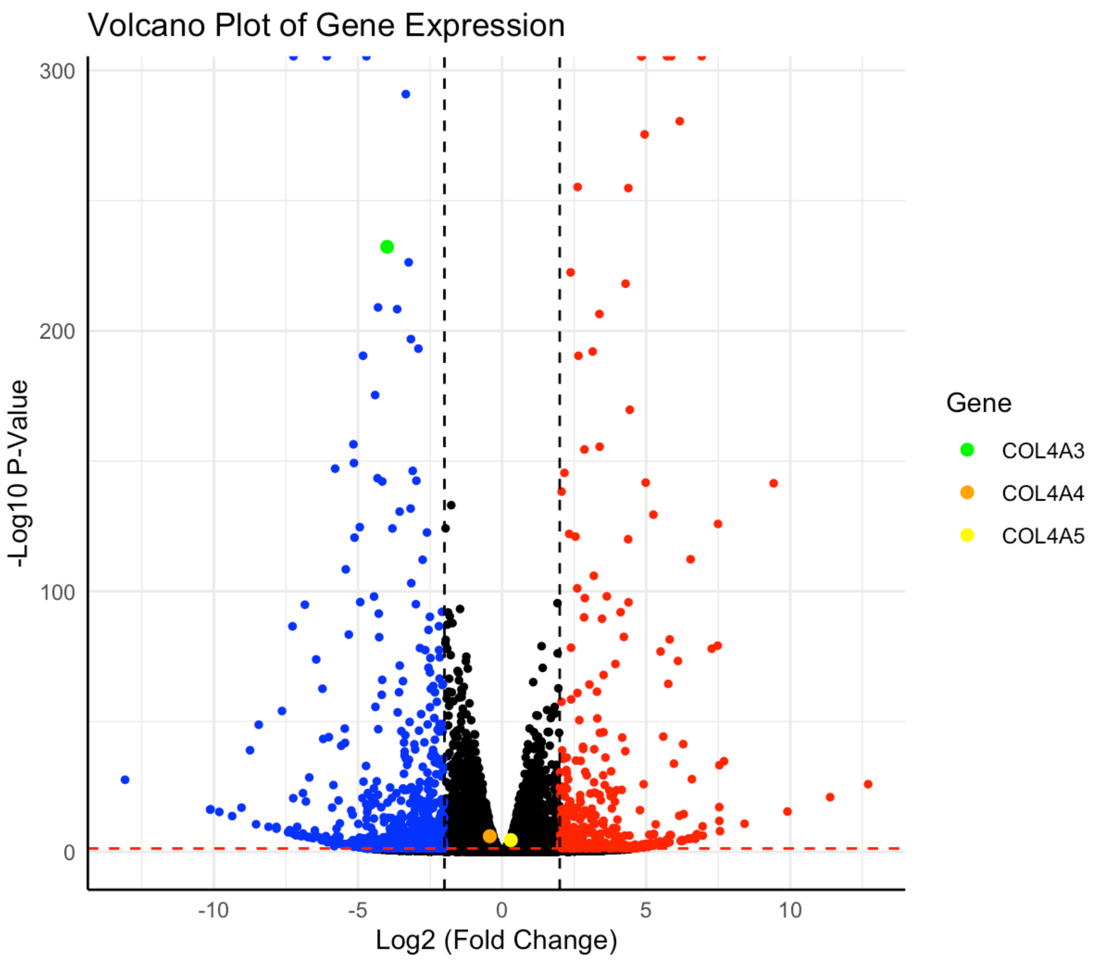

Suppl Figure S1. **Volcano plot of differentially expressed genes (DEG) identified between WT and AS podocytes**. The blue dots denote down-regulated gene expression, the red dots denote up-regulated gene expression, and the black dots denote the gene expression without significant differences.

Method

Transcriptomic data were preprocessed and normalized using *limma* with the *voom* transformation to calculate log2-transformed CPM values. Differentially expressed genes (DEGs) were identified using the *limma* R package, which employs linear modeling and empirical Bayes methods for robust statistical analysis. Significant DEGs were defined based on a p-value threshold of < 0.05 and a fold change threshold of > 2 or < -2. The dataset included 3 WT and 3 AS podocyte samples.

Supplemental data Figure S2.

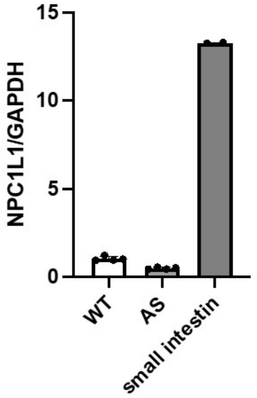

Suppl Figure S2. NPC1L1 mRNA expression levels in immortalized WT and AS podocytes compared to small intestine.

Relative mRNA expression levels (fold change) of NPC1L1 were measured using real-time PCR in WT and AS podocytes, as well as in the small intestine, where NPC1L1 expression has been reported to be abundant. The data indicate that NPC1L1 is expressed

at relatively low levels in both WT and AS podocytes compared to the small intestine. Results are normalized to GAPDH expression and presented as fold change in the graph.
